# Neck cooling provides ergogenic benefits after heat acclimation during exercise in the heat

**DOI:** 10.3389/fphys.2025.1646930

**Published:** 2025-10-17

**Authors:** Guangxia Zhang, Guangjie Xin, Haicheng Li, Pengwei Ma, Haoyan Wang

**Affiliations:** ^1^ College of Physical Education and Health Sciences, Zhejiang Normal University, Jinhua, China; ^2^ Physical Education Department, Guoyuan School, Nantong, China

**Keywords:** cooling strategy, exercise in the heat, heat acclimation, neck cooling, thermal perception

## Abstract

**Background:**

Both heat acclimation and neck cooling have been shown to alleviate heat stress and may help maintain exercise performance in the heat. However, no previous research has been determined their combined effects on thermal perception and physiological responses during exercise in the heat.

**Objective:**

To determine the effects of neck cooling applied before and after heat acclimation on physiological responses, thermal perceptions, and time to exhaustion exercise performance in the heat.

**Methods:**

Fourteen non-heat acclimated men completed a randomized crossover design, involving 10 consecutive days of heat acclimation exercise (HAE) in a controlled heat chamber (35 °C, 30%RH). Participants performed four time-to-exhaustion tests (TTE) with neck cooling (NC) or without (CON) before (Pre-NC and Pre-CON) and after HAE (Post-NC and Post-CON). The TTE test comprised an initial 30 min incremental phase (starting at 50%VO_2max_, increasing 5% every 5 min) followed by a constant workload phase at 70%VO_2max_ until volitional exhaustion. Temperature from tympanic membrane (T_tym_) and neck (T_neck_), heart rate (HR), ratings of perceived exertion (RPE), thermal comfort (TC), thermal acceptability (TA), and thermal sensation (TS) were recorded at 5 min interval.

**Results:**

Following 10 days of HAE, participants exhibited a significant increase in local sweat rate (p = 0.002), and reductions in sweat sodium concentration (p = 0.04) and HR (p = 0.02). TTE performance exhibited significant main effects for both cooling (p = 0.003) and HAE (p = 0.002), while the interaction effect approached significance (p = 0.069). Comparing to the Pre-CON trial, the Post-NC had a significantly greater percentage improvement in TTE (17.3% ± 13.5%) than Pre-NC (3.4% ± 9.9%, p = 0.01) and Post-CON (4.9% ± 13.0%, p = 0.03). Moreover, thermal perception measures revealed significant main effects of cooling (TC: p = 0.01; TS: p < 0.001; TA: p = 0.003) and significant interaction effects (TC: p = 0.036; TS: p = 0.004; TA: p = 0.048), with the Post-NC exhibiting significantly improved TC and reduced TS compared to other groups.

**Conclusion:**

Neck cooling provides ergogenic effects in thermal perceptions and extends a greater improvement in time to exhaustion performance when applied after heat acclimation. These findings suggest that individuals should achieve physiological adaptation to the heat before neck cooling is introduced for perceptual or performance benefits.

## Introduction

Hot and humid environmental conditions present significant challenges for individuals engaging in physical activities. Under such conditions, body core temperature rises rapidly due to metabolic heat production and external thermal load. Previous research has shown that an elevated body core temperature significantly increases thermoregulatory and cardiovascular stress by reducing stroke volume and increasing heart rate, which in turn impairs exercise performance and raises the risk of heat-related illness (Periard et al., 2011; Wingo et al., 2020; Wingo et al., 2005). To mitigate heat stress during exercise in hot environments, heat acclimation and various cooling strategies have been recommended.

Heat acclimation involves a series of physiological adaptations in response to repeated heat exposure through controlled environmental settings (heat acclimation) or natural exposure (heat acclimatization) (Chalmers et al., 2014). Heat acclimation protocol is tailored to an individual’s fitness level and heat acclimation adaptations are impacted by the method and number of heat exposures. Heat acclimation protocols have been categorized into a short-term (<7 days), medium-term (8–14 days), and long-term (>15 days) sessions. Individuals are required to perform exercise for 45–90 min at an intensity ranging from 50% to 75% of their maximal oxygen consumption (Garrett et al., 2011). Consequently, enhanced skin vasodilation and increased sweat sensitivity, which help lower core temperature, are the key physiological changes observed after this process (Chen et al., 2013). A previous meta-analysis also indicated that heat acclimation has a moderate to large beneficial effect on exercise performance in the heat (Tyler et al., 2016). Overall, these adaptations in thermal responses and cardiovascular fitness promote body’s ability to deal with heat stress, thereby maintaining optimal physiological function and exercise capacity.

In addition to heat acclimation, cooling interventions are often used to alleviate hyperthermia during exercise in hot conditions. These interventions include external cooling strategies such as hand cooling (Ruddock et al., 2017), cooling vest (Chaen et al., 2019), and neck cooling (Tyler et al., 2010), as well as internal cooling methods like cold drinks and ice slurry ingestion (Burdon et al., 2013). The efficacy of cooling interventions mainly depends on the duration, the body areas of application and their practicality during exercise (per-cooling) in real-world settings. Recent study suggested that neck cooling is a low-cost, portable, and efficient method for removing heat from the body (Douzi et al., 2020). Neck region is a high alliesthesial thermo-sensitivity area, and previous research indicates that cooling the neck can produce thermal benefits comparable to cooling approximately 60% of the body surface area (Kissen et al., 1971; Shvartz, 1970). However, a recent meta-analysis reported that neck cooling had limited effects on physiological variables during exercise, and improved exercise performance with a small effect size (0.36) (Chan et al., 2015). Previous neck cooling studies have not controlled individual’s heat acclimation status (Galpin et al., 2016; Desai and Bottoms, 2017; Bright et al., 2019; Lee et al., 2014), since both heat acclimation and neck cooling can alleviate heat stress. In addition, no previous study has determined the combine effects of heat acclimation with any cooling regime on physiological responses and exercise performance in the heat. Therefore, the purpose of the present study was to determine the effects of neck cooling on physiological responses, thermal perceptions, and exercise performance prior to, and following a heat acclimation exercise. We hypothesized that both neck cooling and heat acclimation can increase exercise performance. Moreover, when these two strategies are combined, exercise performance would be further improved compared to either strategy alone in a hot environment.

## Materials and methods

### Participants

The sample size was determined using G*Power 3.1. For a two-way repeated measures ANOVA. Based on effect size of 0.3, an α level of 0.05, and statistical power (1-β) of 0.8, a total of 12 participants were required. Finally, fourteen non-heat acclimated men were recruited for this study (age: 22.1 ± 2.5 years, height: 176.0 ± 4.8 cm, weight: 72.9 ± 12.2 kg, and maximal oxygen uptake: 45.4 ± 6.1 mL/kg/min). The experiment was conducted during the spring (April, ambient temperature ∼20 °C), chosen to prevent participants from becoming naturally heat acclimated. The experiment procedures and precautions were instructed to the participants, and informed consent was obtained before any assessment was conducted. Each participant completed a medical history form and a self-screening PAR-Q questionnaire. All participants were free from any disease or exercise injuries that limited ability to exercise. In addition, they were instructed to abstain from alcohol, caffeine-containing beverages, and strenuous exercise 24 h prior to any exercise trial. The study protocol was approved by the University Ethics Committee (#ZSRT2023009) and conducted in accordance with the standards outlined in the Declaration of Helsinki.

### Experimental design

**FIGURE 1:**
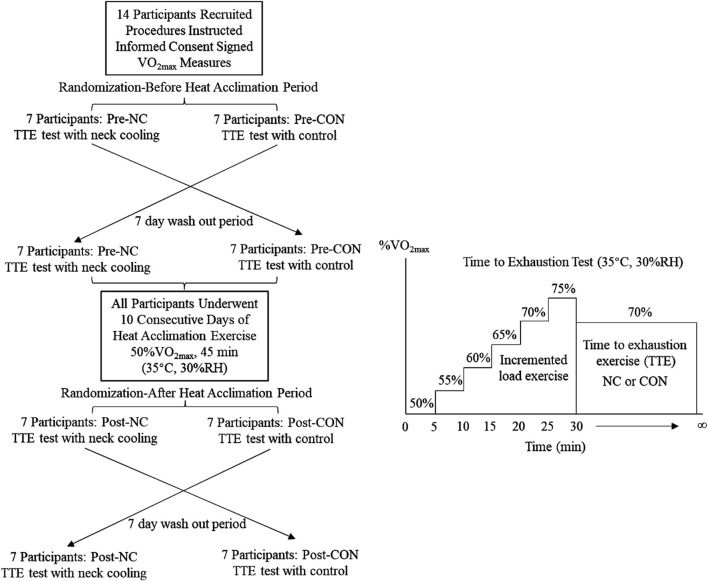
Schematic representation of the experimental design and exercise protocol. Pre-CON, control trial before heat acclimation; Pre-NC, neck cooling before heat acclimation; Post-CON, control trial after heat acclimation; Post-NC, neck cooling after heat acclimation.

The present study employed a randomized cross-over design (Figure 1) and each participant had 15 laboratory visits, including a baseline screening, 10 consecutive days of heat acclimation exercise (HAE), and 4 trials of time to exhaustion test (TTE). Time to exhaustion tests were conducted in a climate-control heat chamber (35 °C, 30%RH) under four experimental conditions: neck cooling before HAE (Pre-NC), control trial before HAE (Pre-CON), neck cooling after HAE (Post-NC), and control trial after HAE (Post-CON). A washout period of 7 days was implemented between the neck cooling trial and control trial. In addition, maximal oxygen uptake (VO_2max_) was assessed using the Bruce treadmill protocol by an indirect calorimetry (CosMed, K5) in a temperate environment. The test started at a low speed and grade, with gradually increased in speed and grade for every 3 min until volitional fatigue was reached. The VO_2max_ values were used to prescribe exercise intensity for subsequent trials (%VO_2max_).

### Time to exhaustion exercise (TTE)

Upon arrival at the laboratory, participants were voided their bladder to provide a urine sample for the assessment of urine specific gravity (USG) using a refractometer (ATAGO, PAL-10s). A USG value below 1.020 was required to maintain euhydration status prior to exercise (Li et al., 2024). Participants with USG exceeding 1.020 were asked to reschedule the visit. Body weight was then measured using a digital scale (TANITA HD-395, precision ±0.1 kg) while participants wore only shorts and a heart rate monitor was worn around the chest (Polar Team-2). Following a 5-min warm-up on the treadmill, participants started TTE test. The TTE test consisted of two phases (Figure 1): 1) a 30 min incremental exercise phase, beginning at 50%VO_2max_ with intensity increasing by 5% every 5 min; 2) a constant-load phase at 70%VO_2max_ until volitionary fatigue was reached. In the neck cooling trials (NC), participants wore a neck cooling collar (Black Ice LLC, United States) during the second phase of the TTE. Neck cooling collar is 375 mm length × 60 mm width × 15 mm thickness, weighs 155 g, and cover the back and the sides of the neck. Neck cooling collar was stored in −80 °C for 24 h and set at temperate environment 10 min before application. In the control trials (CON), no cooling intervention was applied. Heart rate (HR), rating of perceived exertion (RPE, Borg 6–20), neck temperature (T_neck_; Deli DL333380), and tympanic membrane temperature (T_tym_; Braun IRT 6030) were recorded every 5 min. Participants were permitted to drink *ad-libitum* throughout the TTE test. Post-exercise body weight was also assessed to determine body mass loss (BML).

### Heat acclimation exercise (HAE)

The present study implemented a medium-term heat acclimation exercise protocol, in which participants performed treadmill running at 50%VO_2max_, 45 min per day over 10 consecutive days in a heat chamber (35 °C, 30% RH) (Castle et al., 2011). Participants were also required to arrive at the lab in an euhydrated status (USG<1.020) on each day of HAE. During exercise, sweat was collected from the lower back using a 16 cm^2^ absorbent patch (Morris et al., 2013). The skin surface was cleaned with an alcohol spray and dried before the patch was applied. After exercise, sweat sample was extracted from the patch for sweat sodium analysis (Na^+^) using ion-selected probes (AUDICOM AC9900). The local sweat rate (LSR) was calculated as the change in patch weight divided by the product of the patch area and exercise duration (mg/cm^2^/min). T_tym_, HR, and RPE were recorded every 5 min interval throughout each session.

### Thermal perception measures

Subjective thermal perceptions were evaluated using the valid scales (Zolfaghari and Maerefat, 2011). Thermal sensation (TS) scale consists of “+3, +2, +1, 0, −1, −2, −3”, which represent “hot, slightly hot, warm, neutral, slightly cool, cool, and cold” respectively. The thermal comfort (TC) scale consists of “+3, +2, +1, −1, −2, −3”, which represent “very comfortable, comfortable, slightly comfortable, slightly uncomfortable, uncomfortable, very uncomfortable” respectively. Thermal acceptability (TA) scale consists of “+3, +2, +1, −1, −2, −3”, representing “very acceptable, acceptable, slightly acceptable, slightly unacceptable, unacceptable, very unacceptable”. These subjective scales (TS, TC, and TA) were assessed every 5 min interval during TTE test.

### Statistical analysis

Statistical analysis was performed using JMP Pro 16.2.0 software (SAS Inc.), with data reported as mean ± standard deviation. A linear mixed-effects model was applied to analyze physiological changes over the 10-day heat acclimation, with participant ID treated as a random effect, and LSR, HR, T_tym_, sweat sodium concentration as fixed effects. A two-way repeated measures of ANOVA (cooling*HAE) was used to compare TTE performance, physiological responses (fluid balance, HR_mean_, RPE_mean_, △T_tym_, and △T_neck_), and thermal perceptions (TC_mean_, TA_mean_, and TS_mean_). The percentage change in TTE performance (△%TTE) was calculated for Pre-NC, Post-NC, and Post-CON relative to Pre-CON, and one-way ANOVA was applied to compare these differences. In addition, a separate two-way repeated measure ANOVA (cooling*timepoint) was conducted to examine differences in TC and TS across trials before and after heat acclimation trials, respectively, during 35–60 min of the TTE test, as no participant exceeded 60 min in the Pre-CON trial. Tukey’s HSD was performed for *post hoc* pairwise comparisons. Statistical significance was set p < 0.05.

## Results

### Heat acclimation exercise

The linear mixed-effects model analysis indicated that participants who completed the 10 days of HAE exhibited a significant increase in LSR (p = 0.002), along with significant decreases in sweat Na^+^ concentration (p = 0.04) and HR (p = 0.02). However, no significant changes were observed in resting T_tym_ (p = 0.28) or exercise T_tym_ (p = 0.29) over the 10 days of HAE period (Table 1).

**TABLE 1:** Physiological responses across 10-day heat acclimation exercise (n = 14).

	Heart rate (bpm)	LSR (mg/cm^2^/min)	Sweat Na^+^ (mmol/L)	Resting T_tym_ (°C)
Day 1	132 ± 14	3.16 ± 1.08	67.4 ± 20.9	36.6 ± 0.3
Day 2	127 ± 14	3.11 ± 1.60	67.1 ± 20.4	36.5 ± 0.3
Day 3	128 ± 15	3.08 ± 1.16	64.5 ± 21.7	36.5 ± 0.3
Day 4	127 ± 10	3.81 ± 1.66	64.0 ± 23.2	36.5 ± 0.4
Day 5	129 ± 12	3.61 ± 1.33	61.4 ± 22.4	36.6 ± 0.4
Day 6	126 ± 10	4.03 ± 1.67	58.5 ± 18.9	36.6 ± 0.4
Day 7	122 ± 9	3.69 ± 1.86	59.6 ± 18.5	36.6 ± 0.3
Day 8	125 ± 8	4.27 ± 2.04	57.8 ± 13.7	36.5 ± 0.3
Day 9	128 ± 10	4.66 ± 3.36	58.8 ± 14.8	36.7 ± 0.3
Day 10	125 ± 9	4.01 ± 1.96	56.7 ± 16.1	36.6 ± 0.4
P value	0.02	0.002	0.04	0.28

LSR, local sweat rate; Sweat Na^+^, sweat sodium concentration; T_tym_, tympanic membrane temperature.

### Fluid balance and TTE performance

Participants had similar fluid balance in %BML (cooling p = 0.87, HAE p = 0.47, interaction p = 0.54), pre-exercise USG (cooling p = 0.89, HAE p = 0.94, interaction p = 0.34), and post-exercise USG (cooling p = 0.92, HAE p = 0.81, interaction p = 0.08) across trials. Both pre-exercise and post-exercise USG values remained below the critical hypohydration level of 1.020.

**FIGURE 2:**
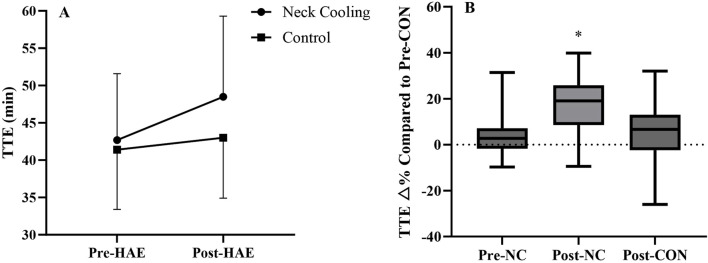
Time to exhaustion performance (TTE) across trials **(A)** and percentage change in TTE by comparing to Pre-CON (TTE △%; **(B)**); Main effect of cooling (p = 0.003), main effect of HAE (p = 0.002), and interaction effect (p = 0.069). *By comparing to Pre-CON, TTE △% in the Post-NC trial was significant different than other trials. Pre-CON, control trial before heat acclimation; Pre-NC, neck cooling before heat acclimation; Post-CON, control trial after heat acclimation; Post-NC, neck cooling after heat acclimation.

TTE performance exhibited significant main effects for cooling (p = 0.003) and HAE (p = 0.002), while the interaction effect approached significance (p = 0.069). Mean TTE performance was completed as follows: Pre-CON (41.4 ± 8.0 min), Pre-NC (42.7 ± 8.9 min), Post-CON (43.0 ± 8.1 min), and Post-NC (48.5 ± 10.8 min, Figure 2A). In addition, compared to the Pre-CON, the percentage improvement in TTE was significantly greater in Post-NC (17.3% ± 13.5%) than Pre-NC (3.4% ± 9.9%, p = 0.01) and Post-CON (4.9% ± 13.0%, p = 0.03, Figure 2B).

### Physiological reponses

Table 2 displays the changes in neck and tympanic membrane temperature responses during the TTE across trials. A two-way repeated measures ANOVA indicated a significant main effect of cooling intervention on neck temperature change (p < 0.001) and a significant main effect of HAE on tympanic membrane temperature change (p < 0.001). However, no significant main or interaction effects were observed for HR (cooling p = 0.82, HAE p = 0.92, interaction p = 0.71) or RPE (cooling p = 0.89, HAE p = 0.26, interaction p = 0.37).

**TABLE 2:** Fluid balance parameters, physiological responses, and thermal perceptions across trials (n = 14) during TTE test.

	Pre-CON	Pre-NC	Post-CON	Post-NC	p _HAE effect_	p _cooling effect_	p _interaction_
%BML	1.11 ± 0.34	1.03 ± 0.42	0.97 ± 0.41	1.02 ± 0.29	0.47	0.87	0.54
Pre-USG	1.013 ± 0.005	1.012 ± 0.005	1.012 ± 0.005	1.014 ± 0.006	0.94	0.89	0.34
Post-USG	1.016 ± 0.005	1.014 ± 0.007	1.013 ± 0.005	1.016 ± 0.007	0.81	0.92	0.08
HR_mean_ (bpm)	160 ± 31	162 ± 27	161 ± 29	160 ± 27	0.92	0.82	0.71
△T_tym_ (°C)	0.7 ± 0.3	0.8 ± 0.3	0.5 ± 0.3	0.5 ± 0.2	**<0.001**	0.12	0.38
△T_neck_ (°C)	0.1 ± 1.8	−5.4 ± 1.3	0.3 ± 1.0	−5.4 ± 1.4	0.51	**<0.001**	0.18
RPE_mean_	15 ± 3	16 ± 2	16 ± 2	16 ± 1	0.26	0.89	0.37
TC_mean_	−1.5 ± 1.2	−1.6 ± 1.4	−1.6 ± 1.3	−0.9 ± 1.1^a^	0.10	**0.01**	**0.036**
TS_mean_	1.8 ± 1.1	1.9 ± 1.2	2.0 ± 1.0	1.1 ± 1.1^a^	**0.01**	**<0.001**	**0.004**
TA_mean_	−1.3 ± 1.3	−1.2 ± 1.5	−1.6 ± 1.4	−0.8 ± 1.1^a^	0.34	**0.003**	**0.048**

%BML, %body mass loss; USG, urine specific gravity; HR, heart rate; RPE, rating of perceived exertion; △T_tym_, change in tympanic membrane temperature; △T_neck_, change in neck temperature; TC, thermal comfort; TA, thermal acceptability; TS, thermal sensation; ^a^, Significant different in Post-NC compared to other groups; Pre-CON, control trial before heat acclimation; Pre-NC, neck cooling before heat acclimation; Post-CON, control trial after heat acclimation; Post-NC, neck cooling after heat acclimation; Bold values indicate p < 0.05.

### Thermal perceptions

**FIGURE 3:**
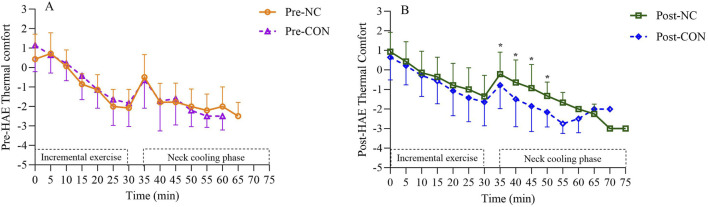
Thermal Comfort (TC) responses at corresponding timepoint before **(A)** and after heat acclimation period **(B)**. *Neck cooling trial was significant different than control trial. Pre-HAE, before heat acclimation period; Post-HAE, after heat acclimation period; Pre-CON, control trial before heat acclimation; Pre-NC, neck cooling before heat acclimation; Post-CON, control trial after heat acclimation; Post-NC, neck cooling after heat acclimation.

Analysis of thermal perceptions revealed that significant main effects of cooling (p = 0.01) and interaction effect (p = 0.036) on TC. In addition, the Post-NC trial resulted in significantly improved TC (−0.9 ± 1.1) during the second phase of TTE compared to Post-CON (−1.6 ± 1.3, p = 0.001), Pre-CON (−1.5 ± 1.2, p = 0.003), and Pre-NC (−1.6 ± 1.4, p = 0.007). As shown in Figure 3, Post-NC had a significant better TC at 35 min (p = 0.04), 40 min (p = 0.002), 45 min (p = 0.001), and 50 min (p = 0.02) compared to Post-CON. No significant differences in TC were observed between Pre-NC and Pre-CON during 35–60 min.

**FIGURE 4:**
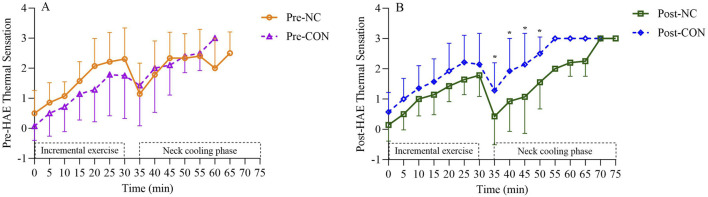
Thermal sensation (TS) responses at corresponding timepoint before **(A)** and after heat acclimation period **(B)**. *Neck cooling trial was significant different than control trial. Pre-HAE, before heat acclimation period; Post-HAE, after heat acclimation period; Pre-CON, control trial before heat acclimation; Pre-NC, neck cooling before heat acclimation; Post-CON, control trial after heat acclimation; Post-NC, neck cooling after heat acclimation.

Similarly, TS exhibited significant main effects for cooling (p < 0.001), HAE (p = 0.01), and interaction effect (p = 0.004). TS was significantly lower in Post-NC (1.1 ± 1.1) compared to Post-CON (2.0 ± 1.0, p < 0.001), Pre-CON (1.8 ± 1.1, p < 0.001), and Pre-NC (1.9 ± 1.2, p < 0.001). Figure 4 indicates that Post-NC had significant lower TS at 35 min (p = 0.004), 40 min (p = 0.001), 45 min (p = 0.001), and 50 min (p = 0.04) compared to Post-CON. No differences in TS were found between Pre-NC and Pre-CON during 35–60 min.

Furthermore, TA showed significant main effects for cooling (p = 0.003) and interaction effect (p = 0.048). TA was significantly higher in Post-NC (−0.8 ± 1.1) compared to Post-CON (−1.6 ± 1.4, p < 0.001), and Pre-CON (−1.3 ± 1.3, p = 0.005).

## Discussion

The present study determined the effects of neck cooling applied both before and after heat acclimation exercise on physiological responses, thermal perceptions and time to exhaustion performance in the heat. Following 10 consecutive days of heat acclimation exercise, participants physiologically adapted to the heat, exhibiting reduced heart rate, increased sweat rate, and decreased sweat sodium concentration. Significant main effects were observed for both cooling and HAE in improving TTE performance. The most substantial improvement occurred when neck cooling was applied after heat acclimation, which appeared to be mediated primarily through enhanced thermal comfort and reduced thermal sensation during exercise in the heat.

### The effects of heat acclimation

The present study employed a medium-term heat acclimation exercise protocol at moderate intensity. Participants exhibited increased sweat rate, decreased heart rate, and reduced sweat sodium concentration over the 10-day heat acclimation period. These responses are consistent with well-established physiological heat adaptations, confirming that our participants successfully adapted to the heat (Periard et al., 2015). In addition, the present study exhibited the main effect of HAE on TTE performance. During the TTE test, participants experienced lower T_tym_ (∼0.2 °C) and thermal sensation, accompanied by a 4.9% improvement in TTE performance, though no significant change in heart rate was detected. In a comparable study, Lorenzo et al., implemented a 10-day protocol consisting 90 min exercise at 50%VO_2max_ under 40 °C conditions. Their regime resulted in an 8% improvement in both exercise performance and VO_2max_ (Lorenzo et al., 2010). This suggests that the observed benefits were likely driven by thermal adaptations rather than cardiovascular adaptations from our HAE protocol. In addition, this smaller improvement in TTE performance may be attributed to the difference in exercise intensity between the acclimation and testing phases. The present HAE was conducted at 50%VO_2max_, while the TTE test required exercise at 70%VO_2max_, creating an intensity gap that likely limited the transfer of acclimation benefits. As emphasized by Sekiguchi et al., more aggressive heat acclimation regimes produce greater physiological and performance adaptations than moderate or self-paced protocols (Sekiguchi et al., 2022). Similarly, Karlsen et al. highlighted that individuals must be expose to sufficiently challenging environmental or exercise stimuli to elicit robust adaptive responses (Karlsen et al., 2015). Thus, these findings underscore the importance of tailoring heat acclimation protocols, including exercise intensity, duration, and environmental conditions to be task-specific, ensuring that the acclimation stress adequately matches the expected performance context.

### The effects of neck cooling

The present study identified a significant cooling effect on TTE performance, which align with previous neck cooling studies demonstrating improvements in TTE performance, self-paced time-trial performance, and repeated-sprint capacity (Tyler and Sunderland, 2011b; Sunderland et al., 2015). The central governor theory and the critical core temperature theory have been widely discussed as potential mechanisms limiting performance in the heat. These studies also indicate that the central governor model is more applicable to self-paced time-trial performances, while the critical core temperature hypothesis is typically relevant to fixed-intensity time-to-exhaustion tests (Tyler and Sunderland, 2011a). Together, these theories reflect two fundamental mechanisms that limit exercise performance in the heat: physiological and psychological factors. Physiological limitations may involve cardiovascular strain, thermal load, energy fuels, hydration status, or a combination of these elements, which dictate the body’s ability to sustain exercise. On the other hand, psychological limitations primarily relate to the perceptions of temperature, such as thermal sensation, thermal comfort, and thermal acceptability (Stevens et al., 2018). In this study, exercise performance was determined by the point of voluntary exhaustion, suggesting the critical influence of perceptual factors on the willingness to maintain exercise intensity. Unlike other cooling strategies (ice slurry, cold water immersion, or cooling garments), which focus mainly on reducing core temperature and cardiovascular strain, neck cooling specifically targets thermal perception. The neck is a region of high thermosensitivity and strong alliesthesia responses, located hear the hypothalamus, and may thereby alleviate subjective thermal discomfort (Cotter and Taylor, 2005). Indeed, our findings demonstrated that neck cooling significantly improved perceived thermal comfort and reduced thermal sensation during exercise.

Notably, when compared to the Pre-CON trial, the Post-NC trial resulted in a 17.3% improvement in TTE performance, whereas Pre-NC yielded only a 3.4% improvement. Castle et al. conducted a study examining pre-cooling applied both before and after a 10-day of heat acclimation period. Their results indicated that pre-cooling had no further performance benefit after heat acclimation. In contrast, the present study demonstrated that exercise performance improved when neck cooling was applied after heat acclimation, with a difference of approximately 14% compared to its application before heat acclimation. Well-documented research has confirmed the beneficial effects of heat acclimation on exercise performance in hot environment. However, the interaction between heat acclimation and cooling strategies appears to influence exercise performance differently. Therefore, future studies should investigate the combined effects of heat acclimation and various cooling interventions to develop more personalized and effective approaches for athletes and recreational individuals in the heat.

### Thermal perceptual responses

Interestingly, out data showed that a differential effect of neck cooling when applied before and after heat acclimation exercise. Although both Pre-NC and Post-NC trials reduced neck temperature to a similar extent (approximately 5 °C), thermal perception responded differently. Neck cooling did not improve thermal perceptions prior to heat acclimation exercise. In addition, thermal comfort, thermal sensation, and thermal acceptability all showed significant cooling and interaction effects, indicating that participants experienced improved thermal comfort and reduced thermal sensation when neck cooling was applied after heat acclimation exercise. To our knowledge, no previous studies have reported such differential outcomes. In the previous neck cooling studies, individuals with higher fitness levels (VO_2max_ ∼45 mL/kg/min vs ∼52 mL/kg/min) seems to derive greater performance benefits from neck cooling intervention than those with lower fitness (Tyler et al., 2010; Tyler and Sunderland, 2011b; Tyler and Sunderland, 2011a; Sunderland et al., 2015). Participants in the present study had moderate fitness (VO_2max_: 45.4 ± 6.1 mL/kg/min) and no recent heat exposure, either in a laboratory setting or natural environment. Psychologically, they exhibited limited tolerance to thermal discomfort rather than reaching a critical core temperature during exercise. After 10 days of heat acclimation exercise, participants experienced heat adaptations, which could reduce thermal strain and improved perceptual tolerance (Tikuisis et al., 2002). Consequently, during the Post-NC trial, participants reported improved thermal perceptions at similar heart rate and RPE compared to Pre-NC. This may also help explain the inconsistent findings across previous neck cooling studies (Cuttell et al., 2016; Bright et al., 2019; Lee et al., 2014; Moss et al., 2021), highlighting the importance of considering acclimation status and fitness level when evaluating neck cooling interventions.

Another potential explanation may be drawn from Bligh’s reciprocal inhibition model of thermoregulation (Bligh, 2006), which posits that warm-sensitive and cold-sensitive neurons within the central nervous system interact through mutual inhibition. Cooling the neck (a region proximal to the hypothalamus) may active cold-sensitive pathways while simultaneously inhibiting heat-loss mechanisms. This antagonistic interaction could result generate conflicting perceptual signals in unacclimated individuals, leading to sustained thermal discomfort. Indeed, participants in the present study reported improved thermal comfort and thermal sensation only when neck cooling applied after heat acclimation, especially during 35–50 min of TTE test. Consequently, our novel findings indicate that heat acclimation should precede the application of neck cooling to optimize both perceptual and exercise performance. Given the high concentration of thermosensitive neurons in the neck region and its proximity to hypothalamic centers, future research should prioritize elucidating the neurophysiological mechanisms through which combined heat acclimation and cooling influence exercise performance, brain activity, and central regulation of thermal perception.

### Strengths and limitations

A key strength of the present study is the well-controlled heat acclimation setting, effectively minimizing the influence of seasonal variation and daily life factors on the outcomes. By acclimating participants exclusively under experimental conditions, we ensured that external variables did not confound the physiological and perceptual responses to intervention. However, several limitations should be acknowledged. First, sweat rate was not measured during the TTE test, which precluded analysis of thermoregulatory adaptations, such as changes in sweat efficiency, across trials. Second, based on previous evidence indicating that neck cooling has minimal influence on core temperature, we did not include continuous core temperature monitoring during TTE or heat acclimation sessions. Lastly, the relatively low humidity level (30% RH) used in this study may enhance evaporative cooling and thus overestimate the efficacy of neck cooling compared to real-world settings where humidity often exceeds 60% (Tsutsumi et al., 2007). Future research should examine the effectiveness of neck cooling under more diverse and ecologically valid environmental conditions, including controlled humidity levels or field-based settings, to improve the generalizability of the findings.

## Conclusion

This is the first study to investigate the effects of neck cooling on physiological responses, thermal perceptions, and exercise performance both before and after a medium-term heat acclimation regime. Our findings make a significant contribution to the understanding of the role of neck cooling in promoting exercise performance in the heat, particularly following acclimation. We demonstrate that a medium-term heat acclimation protocol induces physiological adaptations to the heat, and that an intensity-matched acclimation regimen may further enhance subsequent exercise performance in hot conditions. Furthermore, the results suggest that neck cooling applied after heat acclimation potentially maximize its efficacy on exercise performance. Further studies should employ neurophysiological method, such as electroencephalography, to examine how these interventions modulate brain activity and enhance tolerance during physical or cognitive tasks under thermal stress. Such investigations will provide a more comprehensive understanding of how integrated cooling and acclimation strategies can be optimized to support human performance in hot environments.

## Data Availability

The data presented in this study are available on request from the corresponding author due to privacy and ethical restrictions. Requests to access the datasets should be directed to haoyanw1@zjnu.edu.cn.
